# Cold storage and cryopreservation of tick cell lines

**DOI:** 10.1186/1756-3305-3-37

**Published:** 2010-04-13

**Authors:** Gertrud Lallinger, Erich Zweygarth, Lesley Bell-Sakyi, Lygia MF Passos

**Affiliations:** 1 Technische Universitaet Muenchen (TUM), Munich, Germany; 2 Lehrstuhl für Vergleichende Tropenmedizin und Parasitologie, Ludwig-Maximilians-Universitaet Muenchen, Leopoldstr. 5, 80802, Munich, Germany; 3 The Roslin Institute and Royal (Dick) School of Veterinary Studies, University of Edinburgh, UK; 4 Departamento de Medicina Veterinária Preventiva, Escola de Veterinária- UFMG, Belo Horizonte, Minas Gerais, Brazil

## Abstract

**Background:**

Tick cell lines are now available from fifteen ixodid and argasid species of medical and veterinary importance. However, some tick cell lines can be difficult to cryopreserve, and improved protocols for short- and long-term low temperature storage will greatly enhance their use as tools in tick and tick-borne pathogen research. In the present study, different protocols were evaluated for cold storage and cryopreservation of tick cell lines derived from *Rhipicephalus *(*Boophilus) decoloratus*, *Rhipicephalus *(*Boophilus) microplus, Ixodes ricinus *and *Ixodes scapularis*. For short-term cold storage, cells were kept under refrigeration at 6°C for 15, 30 and 45 days. For cryopreservation in liquid nitrogen, use of a sucrose-phosphate-glutamate freezing buffer (SPG) as cryoprotectant was compared with dimethylsulfoxide (DMSO) supplemented with sucrose. Cell viability was determined by the trypan blue exclusion test and cell morphology was evaluated in Giemsa-stained cytocentrifuge smears.

**Results:**

Cold storage at 6°C for up to 30 days was successful in preserving *R*. (*B.) microplus*, *R*. (*B.) decoloratus, I. ricinus *and *I. scapularis *cell lines; lines from the latter three species could be easily re-cultivated after 45 days under refrigeration. While cell lines from all four tick species cryopreserved with 6% DMSO were successfully resuscitated, the *R*. (*B*.) *decoloratus *cells did not survive freezing in SPG and of the other three species, only the *R*. (*B*.) *microplus *cells resumed growth during the observation period.

**Conclusions:**

This constitutes the first report on successful short-term refrigeration of cells derived from *R*. (*B.) decoloratus*, *R*. (*B.) microplus*, and *I. ricinus*, and use of SPG as an alternative to DMSO for cryopreservation, thus making an important contribution to more reliable and convenient tick cell culture maintenance.

## Background

At present, 879 tick species have been identified worldwide [1] and approximately 10% of these are known to act as vectors of pathogens, leading to disease in domestic animals and humans [2]. In contrast to the number of living tick species, there are relatively few tick cell lines available. The first continuous tick cell lines were established in 1975 [3]; since then, the number of cell lines has increased to over 50, mostly derived from a few economically important ixodid and argasid genera [4-6]. Obligate intracellular bacteria of the genera *Anaplasma*, *Ehrlichia *and *Rickettsia*, and numerous arboviruses have been propagated in tick cell cultures [4]. There is a huge potential to use tick cell lines in a broader range of research areas, from tick molecular biology to host-vector-pathogen relationships. However, one of the constraints to their wider uptake is that the low-temperature storage methods used for tick cells, especially for long-term cryopreservation in liquid nitrogen, are not reliable and do not guarantee successful reestablishment of a resuscitated cell line [7,8]. Indeed some argasid tick cell lines cannot be cryopreserved using established protocols [5,6]. Successful short-term storage has been reported of *Ornithodoros moubata *cell lines at 15°C [5] and of *Ixodes scapularis *cell lines at 12°C [8] and 4°C [9], but a comprehensive study of cell viability following storage was only carried out by the last-mentioned authors.

In the present study, two protocols for the storage and cryopreservation of cell lines derived from three ixodid tick species (*Rhipicephalus *(*Boophilus) decoloratus*, *Rhipicephalus *(*Boophilus) microplus *and *Ixodes ricinus*) were evaluated. Firstly, short-term cold storage of cells in a refrigerator at 6°C for up to 45 days was evaluated, and secondly two cryoprotectants, the commonly-used dimethyl sulfoxide (DMSO) and a sucrose-phosphate-glutamate freezing buffer (SPG) [10], were compared for cryopreservation of the tick cells in liquid nitrogen. The *I. scapularis *cell line IDE8 [8] was included in the experiments as a positive control, as this line has previously been stored successfully using similar refrigeration and DMSO cryopreservation protocols [9].

## Materials and methods

### Tick cell lines and culture conditions

Six embryo-derived tick cell lines were used (Table 1): the *R*. (*B.) microplus *cell lines BME/CTVM2 and BME/CTVM6 [11], the *I. ricinus *cell line IRE/CTVM20 [4], the *I. scapularis *cell line IDE8 [8] and two new cell lines, BDE/CTVM12 and BDE/CTVM14, derived from *R*. (*B.) decoloratus *by a standard technique [7]. Geographic origin of the parent ticks, passage level, culture medium and incubation temperature used for each of the cell lines are presented in Table 1. The complete culture media routinely used for growth of each cell line [8,11,12] were used throughout (Table 1): H-Lac comprises Hank's balanced salt solution supplemented with 0.5% lactalbumin hydrolysate and 20% foetal calf serum (FCS), L-15 comprises L-15 (Leibovitz) medium supplemented with 10% tryptose phosphate broth (TPB) and 20% FCS, and L-15B comprises L-15B medium [13] supplemented with 10% TPB, 5% FCS and 0.1% bovine lipoprotein concentrate (MP Biomedicals). All media were supplemented with 100 IU/ml penicillin and 100 μg/ml streptomycin. Tick cell lines were maintained in flat-sided tubes (Nunc). Medium changes were carried out weekly by removal and replacement of two-thirds of the medium volume. Cultures were passaged at a split ratio of 1:1 at 2-3 week intervals; an equal volume of fresh medium was added to the parent tube, cells were resuspended by gentle pipetting, and half the resultant cell suspension was transferred to a new culture tube (previously conditioned by incubating fresh culture medium therein for several hours) while leaving the remainder in the parent tube for reattachment.

**Table 1:** Tick cell lines used in the low temperature storage experiments: species and geographic origin, passage level, culture and storage conditions.

Cell line	Tick species (geographic origin)	Passage level	Culture medium	Incubation temperature	Storage conditions
BDE/CTVM 12	*R*. (*B.) decoloratus *(Kenya)	10-12	H-Lac	32°C	-196°C
BDE/CTVM 14	*R*. (*B.) decoloratus *(Kenya)	28	H-Lac	28°C	6°C
BME/CTVM 2	*R*. (*B.) microplus *(Costa Rica)	102-104	L-15	28°C	-196°C
BME/CTVM 6	*R*. (*B.) microplus *(Colombia)	174	L-15	28°C	6°C
IRE/CTVM 20	*I. ricinus *(United Kingdom)	118-122	L-15/L-15B^1^	28°C	6°C and -196°C
IDE 8	*I. scapularis *(North America)	103-104	L-15B	32°C	6°C and -196°C

1. Mixture of equal volumes of complete L-15 and L-15B

### Storage under refrigeration

Four cell lines were used: BDE/CTVM14, BME/CTVM6, IRE/CTVM20 and IDE8. For each cell line, cells were harvested by gentle pipetting from 12 to 15 tubes, pooled in a 50 ml centrifuge tube, centrifuged at 400 × g for 10 minutes at 4°C, resuspended in 5-10 ml complete culture medium and counted using a haemocytometer. The cell density was adjusted to 3.3 × 10^6 ^cells/ml and the cell suspension was transferred to a 15 ml centrifuge tube and held in a refrigerator at a temperature of 6°C. For each cell line, after 15, 30 and 45 days the cell suspension was gently resuspended, 3 × 1 ml aliquots were removed and each aliquot was added to 2 ml of the respective complete medium in a culture tube to test for cell proliferation. Thus three replicates were set up for each condition and each cell line. Culture tubes were incubated as described above and maintained for a maximum of 45 days, during which time medium was changed weekly. Subcultures were carried out empirically depending on growth rate as determined by visual inspection of cell density. Day A marks the start of cold storage, and Day 0 marks the day when the cells were removed from the refrigerator for re-cultivation.

### Cryopreservation in liquid nitrogen

Four cell lines were used at cell densities ranging from 2.5 to 3.3 × 10^6 ^cells/ml: BDE/CTVM12, BME/CTVM2, IRE/CTVM20 and IDE8. Two different cryoprotective media were tested on each cell line: (*i*) appropriate complete culture medium containing 6% [v/v] dimethylsulfoxide (DMSO), as described for the cryopreservation of IDE8 cells [9], and supplemented with 1 M sucrose, and (*ii*) a sucrose-phosphate-glutamate freezing buffer (SPG) comprising 74.62 g/l sucrose, 0.517 g/l KH_2_PO_4_, 1.643 g/l K_2_HPO_4_.3 H_2_O and 0.907 g/l potassium glutamate [10].

For each cell line, cells harvested by pipetting from 12 to 15 tubes were pooled, and centrifuged at 400 × g for 10 minutes at 4°C. The cell pellet was resuspended in 5 ml of the respective medium and counted. Half of the cell suspension was held on ice, 2.5 ml of ice-cold medium containing 12% DMSO was added to give a final concentration of 6% DMSO and 1 ml aliquots were transferred to ice-cold 2 ml cryotubes. The other half of the cell suspension was centrifuged as before, the cell pellet was resuspended in 5 ml SPG and 1 ml aliquots were transferred to ice-cold 2 ml cryotubes. Thus three replicates were set up for each condition and each cell line. All cryotubes were frozen at -80°C using a NALGENE^® ^Frosty™ Cryo 1°C freezing container, which resulted in a continuous decrease of temperature at a rate of one degree per minute. After being kept overnight at -80°C, the cryotubes were transferred into a liquid nitrogen tank, where they were stored in the liquid phase for at least seven days.

For resuscitation, the cell suspensions were thawed rapidly by immersion of the cryotubes in a water bath at 37°C. Cells cryopreserved with DMSO were resuspended in 10 ml of the appropriate complete culture medium and were centrifuged at 400 × g at 4°C for 10 minutes. Pellets were resuspended in 1 ml of complete medium and transferred to culture tubes already preconditioned with 2 ml of the same medium for incubation. Cells cryopreserved in SPG were transferred immediately after thawing into culture tubes containing 2 ml of the appropriate complete culture medium. All tubes were incubated and maintained under the respective culture conditions for each cell line (Table 1) for 30 days. Medium changes, subculturing and sampling were done as described above. Day A was the day the cell suspensions were frozen and Day 0 marked the day the frozen cells were resuscitated for re-cultivation.

### Monitoring of cultures

Cultures were monitored weekly, before medium changes, by examination under an inverted microscope. Samples were taken from each culture and, where appropriate, each subculture, to determine the percentage of viable cells by the trypan blue exclusion method [14] on days A, 0, 8, 15, 22 and 30 for both storage conditions and on day 45 for cells stored at 6°C. Furthermore, the morphological appearance of cells was evaluated in Giemsa-stained cytocentrifuge smears.

## Results and discussion

Refrigeration and cryopreservation protocols were tested on cell lines from each of the four tick species. Due to insufficient cell numbers at the start of the study, it was not possible to test both *R*. (*B*.) *decoloratus *and both *R*. (*B*.) *microplus *cell lines by both protocols; therefore one cell line from each species was included in each protocol test.

**Figure 1:**
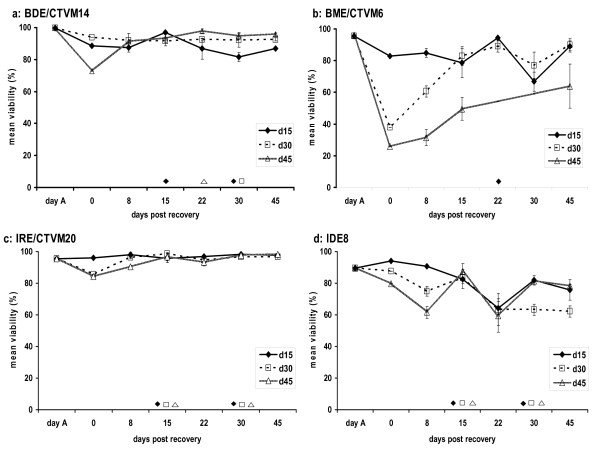
**Viability of tick cell lines after refrigeration**. Viability of tick cell lines (a) BDE/CTVM14, (b) BME/CTVM6, (c) IRE/CTVM20 and (d) IDE8 after storage at 6°C for 15, 30 or 45 days. Symbols indicate when subcultures were carried out and data points are the mean of a minimum of three replicates (parent cultures and, where appropriate, subcultures) for each condition; error bars show standard deviations.

After storage under refrigeration, the BDE/CTVM14 cell line showed viability of over 80% in all replicates at all three storage periods (15, 30 and 45 days), the only exception being on the first day after storage for 45 days. Viability rates remained almost constant during the entire experimental period and parent cultures and subcultures were nearly the same after all three storage periods (Fig 1a). Morphological appearance was almost identical to cells kept under standard culture conditions (without refrigeration), and cells from all three storage periods had multiplied sufficiently to be subcultured at least once during the 45-day observation period (Fig 1a).

The BME/CTVM6 cell line showed good viability after storage at 6°C for 15 days, nearly as high as on day A (Fig 1b), whereas after 30 and 45 days of refrigeration viability rates decreased dramatically to an average of 38% and 26%, respectively. However, within two weeks of cultivation under standard culture conditions samples from day 30 reached viability levels similar to those of samples from day 15 accompanied by slow but steady cell growth, whereas samples from day 45 did not resume growth within the observation period. Subculture was only possible with the cells refrigerated for 15 days (Fig 1b).

The *I. ricinus *cell line IRE/CTVM20 appeared to be unaffected by a refrigeration period of 15 days, as viability over the 45-day period following re-cultivation was similar to that observed on day A (Fig 1c). Furthermore, viability of cells kept for 30 and 45 days at 6°C was slightly lower, but within a week samples reached viability levels comparable to those obtained from cultures at day A or after 15 days of refrigeration (Fig 1c). Samples of cells following all three storage periods appeared morphologically identical to cells kept under standard conditions and had similar growth rates after recultivation, as all could be subcultured successfully twice within the observation period (Fig 1c). In contrast, following recovery from all three storage times the *I. scapularis *cell line IDE8 showed a reduction in cell viability during the 45-day observation period (Fig 1d), although subculture was still possible at fortnightly intervals.

Bastos and co-workers [9] reported that IDE8 cells could be stored successfully at 4°C for up to 60 days. Storage of tick cell lines for longer periods at 12-15°C has been reported [5,8], but maintenance of such temperatures requires specialised cooled incubators which are not available in every laboratory, in contrast to ordinary refrigerators operating at 4-6°C. The ability to store a range of tick cell lines in an ordinary refrigerator for at least 30 days, with the expectation that normal growth will be resumed within 1-2 weeks, will be very useful to cover short periods of inactivity in the laboratory, and preferable to cryopreservation following which some tick cell lines can take up to 6 months to resume normal growth (L. Bell-Sakyi unpublished observations).

**Figure 2:**
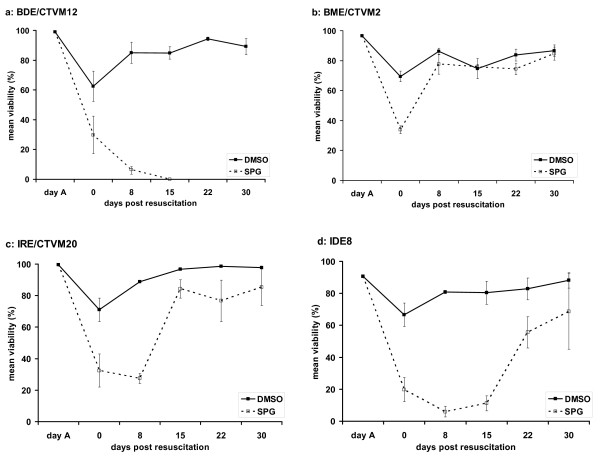
**Viability of tick cell lines after cryopreservation**. Viability of tick cell lines (a) BDE/CTVM12, (b) BME/CTVM2, (c) IRE/CTVM20 and (d) IDE8 after cryopreservation in liquid nitrogen with DMSO or in SPG freezing buffer. Data points are the mean of three replicates for each condition; error bars show standard deviations

Following resuscitation, all cryopreserved cell lines tested showed best results regarding cell viability and cell morphology when DMSO supplemented with sucrose was used as the cryoprotectant (Fig 2). Viability was always higher than 60% on day 0, cell morphology appeared normal at the end of the experiment and all samples had achieved cell densities high enough for subculture after 30 days of incubation. All the tick cell lines tested in the present study have previously been successfully cryopreserved with 10% DMSO [[8]; L. Bell-Sakyi unpublished data]. It is not known whether the lower concentration of 6% DMSO used in the present study and previously [9] resulted in higher or lower initial cell viability following resuscitation compared with 10% DMSO; insufficient total cell numbers precluded inclusion of a 10% DMSO protocol in the present study. However, viability of all four cell lines in the present study at day 30 after resuscitation was over 80%, compared to around 75% achieved by IDE8 cells previously [9]; this improvement may have been due to the inclusion of 1 M sucrose in the cryopreservation medium.

The use of SPG as a cryoprotectant for tick cells led to disappointing results. After resuscitation, BDE/CTVM12 cells all died within two weeks (Fig 2a) in contrast to BME/CTVM2 (Fig 2b), which appeared to be the cell line least affected by cryopreservation in SPG as the cells recovered rapidly and resumed growth. IRE/CTVM20 (Fig 2c) and IDE8 (Fig 2d) also survived cryopreservation in SPG and subsequent resuscitation, although initially their viability was much lower than cells cryopreserved with DMSO, and they did not resume growth during the observation period. This was not surprising, because SPG was originally devised for the preservation of rickettsia [10] and not for tick cells. Nevertheless SPG was included in the present study because it has been used to preserve obligate intracellular rickettsial organisms such as *Rickettsia rickettsii *[10] and *Ehrlichia ruminantium *[15] which can be propagated in some of the tick cell lines examined [11,16]. Moreover, in contrast to crypreservation with DMSO, the components of SPG are themselves not toxic for cells *in vitro *or *in vivo*, and FCS is not an essential ingredient, making this buffer more suitable as a cryoprotectant for material to be used in future vaccine development studies. Use of SPG as a cryoprotectant for argasid tick cells which cannot be successfully frozen with DMSO [5,6] should also be evaluated.

## Conclusions

The results presented here show that cell lines derived from the ixodid tick species *I. ricinus*, *R. (B.) decoloratus *and *R. (B.) microplus *can be successfully stored under refrigeration for at least 30 days. Furthermore, as an alternative to using DMSO as a cryoprotectant, *R. (B.) microplus *cells can be cryopreserved in a sucrose-phosphate-glutamate freezing buffer which allows culture propagation after resuscitation. These protocols will help to extend the use of tick cell lines as tools for tick-borne pathogen research by improving the ease and reliability of their storage at low temperatures.

## Competing interests

The authors declare that they have no competing interests.

## Authors' contributions

GL conducted all laboratory work and contributed to drafting the manuscript. LB-S provided the *Rhipicephalus *spp and *Ixodes ricinus *cell lines and critically revised the manuscript. EZ was involved in critically revising the manuscript for intellectual content. LP developed the conception and design of the study and was involved in drafting the manuscript. All authors have given final approval of the version to be published
